# Ultrasonic Melt Processing: Progress, Applications, and Future Directions

**DOI:** 10.3390/ma18030522

**Published:** 2025-01-23

**Authors:** Shuang Yang, Yu Weng, Qin Zhao, Gang Wu, Zhian Deng, Ling Qin

**Affiliations:** 1 College of Petroleum Engineering, Xi’an Shiyou University, Xi’an 710065, China; 210103@xsyu.edu.cn (S.Y.); wugang@xsyu.edu.cn (G.W.); 2 Xinzhou Branch, PetroChina Coalbed Methane Co., Ltd., Xinzhou 036600, China; zhaoqin128@petrochina.com.cn; 3 School of Energy Resource, Quanzhou Vocational and Technical University, Quanzhou 362000, China; dza--062826@163.com; 4 Centre of Innovation for Flow Through Porous Media, Department of Petroleum Engineering, University of Wyoming, Laramie, WY 82071, USA

**Keywords:** ultrasonic melt processing, synchrotron X-ray imaging, in situ, high-fidelity modeling, ultrasonic bubble, acoustic streaming

## Abstract

Ultrasonic melt processing (UMP) has garnered significant attention from both academic and industrial communities as a promising solution to critical challenges in the metal casting industry. This technique offers a clean, environmentally friendly, and energy-efficient approach to improving melt quality and achieving structural refinement. However, due to the opaque nature of metals, understanding the fundamental mechanisms governing the interactions among ultrasonic bubbles, acoustic streaming, and the melt remains still challenging. This review traces the evolution of UMP research, from its inception in the mid-20th century to recent advancements, with particular emphasis on the application of state-of-the-art synchrotron X-ray imaging and computational modeling. These approaches have been instrumental in unraveling the complex, multiscale dynamics occurring across both temporal and spatial scales. Key findings in various metallic alloy systems are critically reviewed, focusing on new insights into cavitation bubbles, acoustic streaming, and the interactions of growing solid phases in different alloys. Additionally, the review discusses the resulting phenomena, including grain refinement, fragmentation, and the mitigation of solidification defects, in detail. The review concludes by identifying critical research gaps and emerging trends, underscoring the indispensable role of in situ studies and robust theoretical frameworks in advancing UMP. These developments are poised to reshape the future of innovation in materials science and engineering.

## 1. Introduction

Ultrasonic melt processing (UMP) is an advanced technique in materials science that uses high-frequency sound waves to influence the behavior of materials in their molten state [1,2,3]. UMP has gained significant attention for its ability to refine microstructures, enhance material properties, and improve the quality of metallic alloys [4]. The properties and behaviors of molten metals are determined by factors such as temperature, pressure, and the interactions between atoms and other elements in the liquid phase, which can be influenced by external fields such as ultrasonic waves [5,6,7]. While the fundamental understanding of liquid metal processing has been well established over decades, the application of ultrasonic waves to melt processing presents unique opportunities and challenges due to the complex physical phenomena involved, including cavitation, acoustic streaming, and the microstructural evolution [1,7,8,9,10].

Historically, UMP has been explored as a method to refine grain structures, degas molten metals, and mitigate defects during solidification, all of which are essential for producing high-performance alloys [11,12]. The technique is particularly valuable for manufacturing advanced metallic materials with improved mechanical properties, such as enhanced strength, ductility, and resistance to fatigue. Since its first application in the mid-20th century, UMP has evolved significantly, with early research focusing on basic principles and experimental setups, followed by advancements in ultrasonic technology, including the use of high-intensity and high-frequency ultrasound waves [12,13].

X-rays have been used for over a century to observe and analyze the internal structures of metals and other opaque materials [14,15]. Since the mid-1990s, high-brilliance X-rays and advanced instrumentation have been made available to materials scientists through the development of third-generation synchrotron X-ray facilities worldwide. These technological advancements have enabled metals to be studied in situ during solidification processes, where the dynamics of microstructure evolution, defect formation, and phase transformations can be monitored in real-time. By combining high-resolution synchrotron X-ray imaging and diffraction techniques, detailed insights into the internal behavior of metals during solidification have been obtained, providing crucial data for the development of new alloys and the optimization of manufacturing techniques [16,17]. The ability to observe and analyze material behavior under operating conditions has significantly facilitated advancements in materials science, leading to the creation of stronger, more durable, and functional metallic components [18,19,20]. The integration of computational models and high-fidelity experimental methods has significantly advanced the understanding of ultrasonic effects on melt dynamics, making it possible to optimize processing conditions for various alloy systems [21,22].

This review systematically examines the evolution of ultrasonic melt processing from its inception to the present day, focusing on key milestones and developments in the field. It first introduces the fundamental principles of UMP, including the mechanisms of ultrasonic cavitation and acoustic streaming in molten metals. The integration of experimental techniques such as high-speed imaging and in situ diagnostics is explored as a powerful tool for investigating the dynamic behavior of liquid metal systems during UMP. In addition, this review discusses the effects of UMP on grain refinement [23,24,25], degassing [10,26,27,28], and defect mitigation [29,30], along with the implications for different metallic alloys. Finally, it outlines emerging research trends, identifying critical gaps in knowledge and emphasizing the need for more advanced in situ characterization techniques and theoretical models to better understand and harness the full potential of UMP for industrial applications.

## 2. A Brief over Review of the Historical Development of the Melt Processing

**Figure 1:**
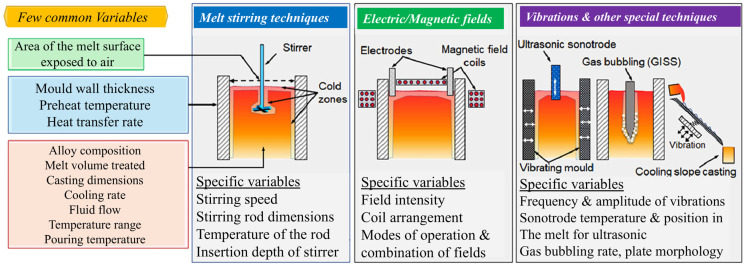
Schematic diagram and brief introduction of five commonly used melt processing technologies [39].

The history of melt processing dates back thousands of years, with its roots in early human efforts to manipulate materials through heating and shaping. The earliest known use of melt processing can be traced to the ancient civilizations, where metals like copper and bronze were melted in primitive furnaces and poured into molds to create tools, weapons, and jewelry [31,32]. This early form of casting allowed for the production of complex shapes that were otherwise difficult to achieve using traditional forging methods [33]. As industrialization progressed, the methods of melt processing became more sophisticated. During the 19th century, the development of modern casting techniques such as sand casting and metal mold casting revolutionized manufacturing, making it possible to produce large quantities of metal parts with greater precision. The advent of the electric arc furnace in the early 20th century provided a more efficient way to melt metals, leading to significant advancements in steel production and the development of various alloys. Throughout the 20th century, the rise in plastics and polymers introduced new challenges and innovations in melt processing. Injection molding became a popular method for producing plastic parts, allowing for rapid and cost-effective production of a wide range of products [34]. The continued development of advanced technologies, such as computer-aided design (CAD) and additive manufacturing, has further expanded the capabilities and applications of melt processing in industries ranging from aerospace to automotive, electronics, and healthcare. Today, melt processing remains a cornerstone of modern manufacturing, with ongoing innovations driving improvements in material properties, process efficiency, and sustainability [31,35,36,37,38]. The following is a detailed explanation of a major melt processing technology. The most common and effective methods are (1) Electromagnetic Stirring (EMS), (2) Melt Stirring, (3) Vibration-Assisted Melt Processing and (4) ultrasonic melt processing, as shown in Figure 1 and described briefly in this section.

### 2.1. Electromagnetic Stirring

Electromagnetic Stirring (EMS) is a technique that uses a magnetic field to induce motion within molten metal, promoting better flow and mixing. This method relies on the interaction between the electromagnetic field and the induced electric currents in the molten metal, generating a Lorentz force that stirs the melt. EMS was first introduced by N.G. Chetverikov and his colleagues in the 1970s [40,41,42] and has undergone significant refinements for various metallurgical applications [43,44]. The key benefits of EMS include enhanced melt homogeneity, reduced segregation, and improved microstructure during solidification. By improving mixing, EMS ensures a more uniform distribution of alloying elements, which reduces the formation of defects such as porosity and segregation [45]. This is especially valuable for high-performance alloys like aluminum, titanium, and steel, which are prone to casting defects [46,47]. EMS also accelerates cooling during solidification by promoting uniform heat dissipation, preventing the formation of coarse grain structures and enhancing the mechanical properties of the final casting, such as strength, toughness, and ductility. Additionally, EMS helps reduce gas porosity and prevents oxide film formation, improving the overall quality of cast components [48,49,50,51].

### 2.2. Melt Stirring

Melt Stirring is a processing technique that involves mechanically agitating a molten metal or alloy to enhance its properties and improve processing outcomes [52]. By introducing stirring forces through mechanical or electromagnetic means, melt stirring promotes better homogeneity of the melt, ensuring the uniform distribution of alloying elements and improving the overall material quality. This technique helps in the refinement of the microstructure, reducing the formation of defects such as porosity and segregation. It also enhances the removal of impurities, as stirring facilitates the removal of gas pockets and solid inclusions from the melt [53]. Additionally, melt stirring improves heat transfer, leading to more uniform cooling and solidification. Widely applied in metal casting, alloy production, and manufacturing industries like aerospace, automotive, and electronics, melt stirring plays a key role in improving the performance and reliability of materials used in demanding applications [54].

### 2.3. Vibration-Assisted Melt Processing

Vibration-Assisted Melt Processing (VAMP) utilizes controlled mechanical vibrations to improve the processing of molten metals [55,56]. By applying vibrations at specific frequencies and amplitudes, this technique influences the molten metal’s behavior, enhancing flow characteristics and promoting a more uniform melt. Vibrations help break up gas bubbles, reduce slag inclusions, and improve the distribution of alloying elements, leading to better microstructural refinement. The development of VAMP began with early research into the effects of mechanical vibrations on molten metals, particularly in casting and refining applications. Over time, it became clear that vibrations significantly improve melt homogeneity and quality, minimizing defects like segregation and porosity [57]. One of VAMP’s primary advantages is its ability to eliminate or reduce inclusions and undesirable phases in the molten metal. These inclusions, often caused by contaminants or improper melting, can lead to defects such as reduced mechanical performance or poor surface finish. Vibrations disperse these inclusions more evenly in the melt or help them dissolve, resulting in a finer, more homogeneous microstructure in the solidified metal [58]. Additionally, VAMP enhances the solidification process by improving heat transfer between the molten metal and the mold, which accelerates cooling rates and produces more refined grain structures [59]. The vibrations also improve the fluidity of the molten metal, which is particularly beneficial for casting complex shapes or high-performance alloys. The result is casting with superior mechanical properties, reduced defects, and improved overall performance. VAMP is widely used in industries that involve casting high-performance materials, including aerospace alloys, automotive components, and advanced manufacturing sectors, where its ability to enhance material quality and performance is crucial.

### 2.4. Gas Bubbling-Assisted Melt Processing

Gas Bubbling-Assisted Melt Processing involves injecting gas bubbles into molten metals or alloys to enhance material properties and processing [60,61]. The introduction of gases like nitrogen, hydrogen, or inert gases improves material purity by removing harmful gases and solid inclusions. It also enhances the flowability of the melt, which aids in precise forming processes. Additionally, the bubbles promote grain refinement by encouraging uniform nucleation, improving the material’s mechanical properties. The process improves thermal conductivity, leading to more uniform cooling and reduced thermal stress, while also promoting the uniform distribution of alloying elements, preventing segregation. This technique is widely used in metal casting, alloy production, aluminum refining, and high-performance materials in industries like aerospace, automotive, and manufacturing, optimizing both macroscopic and microscopic properties [62].

### 2.5. Ultrasonic-Assisted Melt Processing

**Figure 2:**
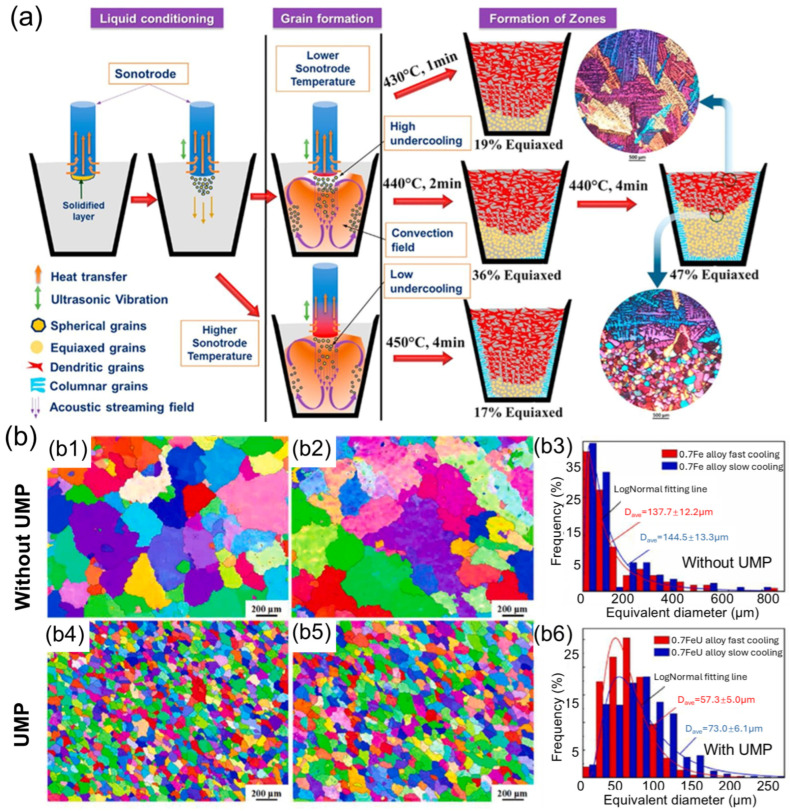
(**a**) Various key mechanisms involved in UMP and (**b**) the EBSD mapping and grain size distribution under various conditions, including (**b1**,**b4**) 0.7 Fe and 0.7 FeU alloys with a fast cooling rate, (**b2**,**b5**) 0.7 Fe and 0.7 FeU alloys with a slow cooling rate, and (**b3**,**b6**) the corresponding grain size distributions [15].

Since the 1930s, ultrasonic melt processing (UMP) has attracted considerable attention from both academic researchers and industry professionals. It utilizes high-frequency sound waves to modify the behavior of molten metals, offering distinct advantages over other melt processing techniques [63,64,65]. By introducing ultrasonic waves into the molten metal, this method breaks up gas bubbles, reduces slag inclusions, and promotes a more uniform distribution of alloying elements. UMP is especially effective in reducing gas porosity and improving the overall quality of the material [4,66]. Figure 2 illustrates the typical process steps of UMP and its impact on grain size distribution.

One of the key advantages of UMP, compared to techniques like EMS and Vibration- VAMP, is its ability to achieve finer control over the microstructure. The high-frequency sound waves generate small-scale cavitation, which can help refine the grain structure more effectively than mechanical stirring methods. This leads to enhanced material properties such as increased strength and ductility, with a finer and more homogeneous microstructure. Unlike EMS, which primarily relies on magnetic fields and the Lorentz force to induce stirring, UMP provides a more direct influence on the melt’s characteristics. EMS can be more energy-intensive and may have limitations in its ability to break up gas bubbles or handle complex melt flow behaviors. In contrast, UMP can target specific areas of the melt with precise control, making it highly effective for reducing defects like gas entrapment and ensuring uniform alloy distribution. When compared to VAMP, which applies mechanical vibrations to improve melt uniformity, UMP offers the added benefit of working at much smaller scales, providing greater refinement at the microstructural level. While VAMP is effective for improving the overall flow and reducing larger inclusions, UMP excels in achieving fine-scale uniformity and reducing small-scale defects, such as fine gas bubbles and slag inclusions. Moreover, UMP can also be integrated with additive manufacturing technology [67,68,69]. Over the past five years, we have conducted extensive research on various materials in the field of ultrasonic processing [70,71,72,73,74,75].

## 3. The Fundamental Issues of Ultrasonic Melt Processing

However, one of the fundamental challenges in this process lies in the inherent opacity of metals in their molten state, which prevents direct observation of the underlying mechanisms occurring during ultrasonic treatment. At the microscopic scale, the cavitation phenomena driven by ultrasound—where bubbles form and collapse in the molten metal—are not easily visualized due to the opaque nature of the liquid metal. These microscopic events are crucial for generating shear forces and local temperature gradients that impact the microstructure, but understanding the exact nature of these interactions requires advanced indirect techniques like X-ray imaging or modeling, which are limited by resolution and accessibility. On the mesoscale, while the cavitation bubbles interact dynamically with the molten material, the lack of visual access makes it difficult to study how these interactions influence the material properties in real time. The collapse of these bubbles generates high-intensity localized forces that are believed to refine grain structures and improve material homogeneity, but these effects cannot be directly observed in the melt. As a result, the efficiency and outcome of the ultrasonic treatment depend largely on theoretical models and indirect measurements. The variation in bubble collapse intensity and spatial distribution also adds complexity, as this variability can affect the consistency of the treatment, but again, it is challenging to correlate these factors with actual material changes without visual confirmation. At the macroscopic level, the success of ultrasonic melt processing is contingent on maintaining dispersion and stability within the molten metal. Ultrasound can break up large aggregates and prevent phase segregation, but the lack of real-time observation complicates the optimization of parameters such as ultrasound frequency, amplitude, and pulse duration. Furthermore, the challenge of scalability becomes more pronounced when transitioning from small-scale experiments to industrial applications, as uniform cavitation and heat dissipation are harder to control in larger systems. The inability to directly observe these processes limits the ability to refine and fine-tune ultrasonic melt processing, which poses a significant challenge for improving reproducibility and achieving consistent, high-quality material outcomes on a larger scale.

## 4. Experimental Techniques for Studying Ultrasonic Melt Processing

### 4.1. A Brief Overview of the Development of Synchrotron X-Rays

Synchrotron X-ray technology has undergone significant development since its inception in the 1940s [76,77]. Initially, synchrotron radiation was discovered as a byproduct of particle accelerators used in high-energy physics [77]. Scientists soon realized its potential as a powerful tool for material characterization due to its unique properties, including high brightness, tunable wavelength, and the ability to probe deep into materials. In the early years, synchrotron radiation was primarily used in basic research, such as in studying the atomic structure of materials and fundamental physical processes. As synchrotron facilities evolved, their applications expanded beyond basic research into advanced fields like materials science, biology, and chemistry. By the 1980s and 1990s, the advent of third-generation synchrotron sources represented a significant breakthrough. These advanced sources generated intense X-ray beams with exceptional focus and precision, allowing researchers to investigate the internal structures of materials at unprecedented resolutions [15,78].

**Figure 3:**
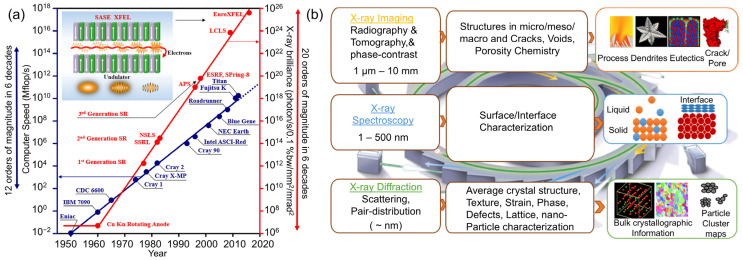
(**a**) The historical evolution of X-ray sources, highlighting advancements in X-ray brilliance over time. (**b**) A selection of pivotal synchrotron X-ray-based in situ and operando characterization techniques for metals, showcasing their spatial and temporal scales, key capabilities, and representative applications [84].

Synchrotron X-ray imaging, in particular, became a key application in materials science. It allows for the non-destructive observation of a material’s internal features without altering its properties. One of the most notable techniques is X-ray tomography, which provides 3D images of a sample’s internal structure. Synchrotron X-ray imaging has been used extensively to study a wide range of materials, from soft biological tissues to hard metals and composites, making it an indispensable tool in fields like engineering, medicine, and environmental science. In metallurgy, synchrotron X-ray imaging has become an indispensable tool for investigating metals and alloys. It provides real-time insights into microstructural changes during critical metallurgical processes such as high-temperature treatments, deformation, and solidification. For example, during alloy solidification, synchrotron X-ray imaging can unveil the formation and evolution of internal defects such as porosity, voids, and cracks. This information is instrumental in optimizing process parameters and enhancing product quality. Additionally, synchrotron X-ray imaging is extensively employed to study phase transformations, grain structures, and interfacial interactions in metallic materials. These insights enable metallurgists to design materials with superior performance characteristics, including improved mechanical properties, enhanced corrosion resistance, and optimized functionality. Figure 3 illustrates the historical evolution of X-ray sources, showcasing the transformative advancements from conventional X-ray tubes to modern synchrotron light sources and X-ray free-electron lasers. It highlights the exponential increase in X-ray brilliance over time and its profound implications for high-resolution material characterization. The figure also provides an overview of key synchrotron X-ray-based in situ and operando characterization techniques for metals, highlighting their spatial and temporal resolution scales, advanced capabilities—such as imaging, diffraction, and spectroscopy—and notable applications in understanding phase transformations, defect dynamics, and stress evolution across multiple length scales. We have conducted extensive research utilizing synchrotron and laboratory-based X-ray methods [79,80,81,82,83]. In the following sections, we will systematically explore the advancements in synchrotron X-ray technology in ultrasonic melt processing.

### 4.2. Scientific Focuses of the Research on Ultrasonic Melt Processing

#### 4.2.1. Experiment Setup for Studying Ultrasonic Melt Processing

**Figure 4:**
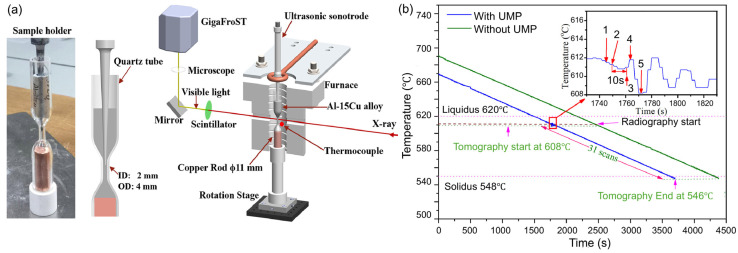
Schematic of the experimental setup for radiography and tomography under ultrasound: (**a**) custom quartz sample holder and furnace; (**b**) temperature profiles and operational details during the experiment (1—sonotrode move down; 2—UMP and radiography start; 3—UMP and radiography stop; 4—sonotrode remove; 5—tomography start) [85].

Figure 4 illustrates a typical experimental setup designed for in situ synchrotron imaging and tomography within the melt. The sample holder, made from a quartz tube with variable cross-sections, featured a central section with a 2 mm inner diameter, a 4 mm outer diameter, and a 10 mm height, allowing X-ray transmission for imaging. A thermocouple was placed near the midpoint of the tube to record temperature, as shown in Figure 4a. Surrounding the sample holder was a furnace that enabled remote and precise temperature control, facilitating alloy melting and experiments conducted at a specific cooling rate. To ensure reproducible solidification conditions, temperature calibration was performed and repeated multiple times. Real-time radiography was used to determine the temperature, recorded by the thermocouple, at which dendritic grains first appeared in the field of view (FOV), triggering image recording at that precise moment. This protocol was consistently applied to all samples during radiography acquisitions. It is important to note that the ultrasound sonotrode tip had to be removed during the imaging period, as tomography acquisition requires sample rotation. For the alloy systems with different melting points, replacing the heating element was necessary to ensure the complete melting of the metal.

#### 4.2.2. Ultrasonic Bubble in Melt

**Figure 5:**
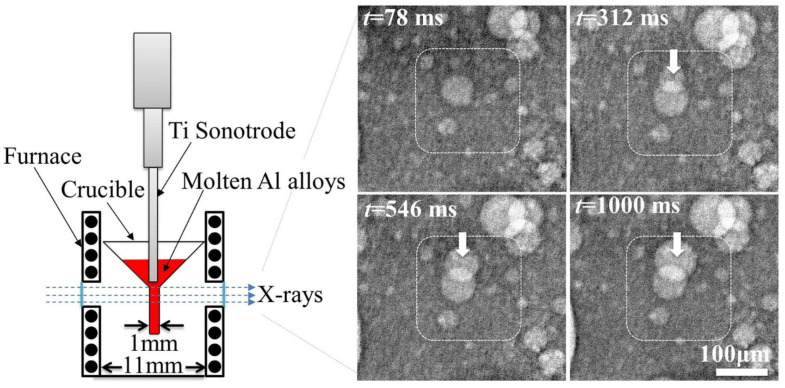
Experimental setup and X-ray imaging, showing the nucleation and growth dynamics of ultrasonic bubbles in a molten Al–10 wt.% Cu alloy [88].

When the FOV is positioned close to the sonotrode tip, cavitation bubbles may become visible. The distribution and dynamic behavior of these bubbles significantly influence the subsequent solidification process of the alloy [70,86,87]. Xu et al. [88] used in situ synchrotron X-ray radiography to investigate the dynamic behavior of ultrasonic bubbles generated by ultrasound in melt, as shown in Figure 5. The size distribution, growth rate, and average bubble radius were measured under conditions of an 800 W/cm^2^ acoustic intensity and a peak acoustic pressure of 4.5 MPa (approximately 45 atm). The results revealed that the bubbles followed a log-normal size distribution, with an average radius of 15.3 ± 0.5 μm. Building on this, Mi et al. [89] further reported an ultrafast synchrotron X-ray imaging study (up to 271,554 frames per second) at the Advanced Photon Source, Lemont, IL, USA, to explore the dynamics of ultrasonic bubbles in a Bi-8% Zn alloy. For the first time, chaotic bubble implosions and dynamic oscillations in both liquid and semisolid metals were captured. However, due to the limited FOV, this study was unable to capture the shock waves generated by the implosion of the bubbles. This progression of research highlights the complexity of bubble behavior in molten metals and emphasizes the importance of further investigation to capture more dynamic events, such as shock wave propagation, that are crucial to understanding the full impact of ultrasound on the solidification process.

**Figure 6:**
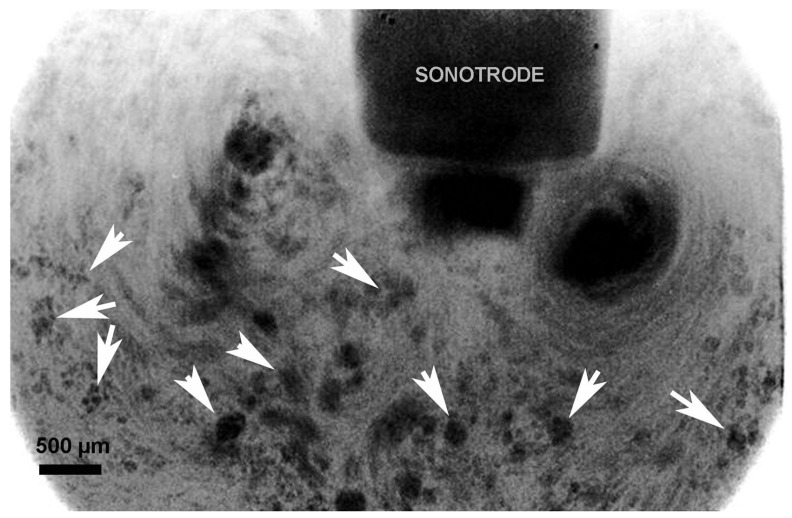
The acoustic streaming pattern (indicated by arrows), observed during synchrotron experiments with a Sn–30 wt.% Cu alloy under 100 W of applied acoustic power, revealing two nearly symmetrical clockwise vortices below the sonotrode tip [28].

The second physical phenomenon, acoustic streaming, can be observed using synchrotron X-ray imaging. Originating from the cavitation zone, this phenomenon manifests as large-scale fluid motion driven by acoustic energy generated during cavitation events. As shown in Figure 6, a typical flow pattern includes a downstream flow emanating from the cavitation zone and two symmetric recirculation vortices on either side. These vortices arise from the intricate interactions between acoustic waves, cavitation bubbles, and the surrounding fluid, leading to localized pressure gradients and fluid movement.

#### 4.2.3. Interaction Between Ultrasound and Solid Phase During Solidification

**Figure 7:**
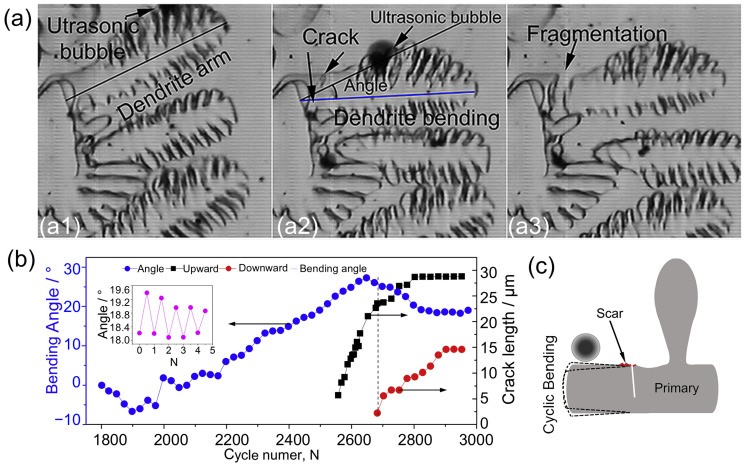
(**a**) Deformation and crack formation at the base of a dendrite after interaction with an oscillating bubble: (**a1**) ultrasound induces oscillation near the dendrite arm; (**a2**) a crack forms; (**a3**) fragmentation occurs. (**b**) Progression of crack length in relation to the number of ultrasonic bubble oscillation cycles for the fragmented dendrites. (**c**) Schematic representation of the cyclic deformation effect caused by the oscillating bubble [90].

Before the advent of widely accessible synchrotron X-ray imaging, the SCN-5 wt.% camphor transparent alloy was commonly used as the primary experimental material for real-time observations. Its transparency enabled high-speed filming under general illumination, making it an effective tool for studying dynamic processes. Using SCN-5 wt.% Camphor transparent alloy as a case study, Wang et al. [90] performed a detailed investigation of dendritic fatigue fragmentation under an ultrasonic field using high-speed cameras in Figure 7. The dynamic interactions between oscillating ultrasonic bubbles and both primary and secondary dendrites were captured in their study. Extensive data from high-speed images revealed that when an ultrasonic bubble became trapped in an interdendritic region, its oscillations induced the cyclic bending of the dendritic arms. This cyclic motion led to the initiation of a fatigue crack at the base of the dendrite arm, which then propagated along the structure, ultimately causing dendrite fragmentation—a phenomenon known as dendritic fragmentation. While these findings provide significant insights into the mechanisms of dendritic fragmentation, it is important to note that the physical properties of SCN-5 wt.% camphor alloy differ considerably from those of metallic alloys, potentially limiting its broader applicability as a model for metal systems.

**Figure 8:**
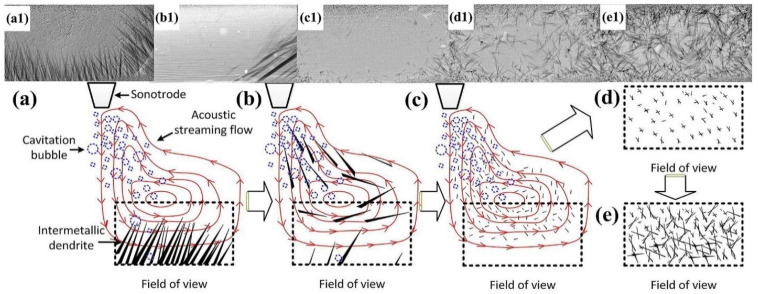
A schematic and X-ray image depicting the process of the microstructural refinement of primary intermetallic dendrites under UMP: (**a**) UMP induces acoustic cavitation and streaming flow; (**b**) the combined effects of cavitation and streaming flow cause the fragmentation of intermetallic dendrites; (**c**) continuous circulation of the fragments by the acoustic flow reintroduces them into the cavitation zone, resulting in further fragmentation and the formation of numerous small pieces; (**d**) once UMP ceases, the remaining smaller fragments sink and develop into equiaxed dendrites; (**e**) as these equiaxed dendrites grow, they collide with one another, leading to further refinement of the structure (Arrows indicate the direction of flow), panels (**a1**–**e1**) present the corresponding X-ray images for each stage (**a**–**e**) [91].

Compared to the SCN-5 wt.% camphor transparent alloy, intermetallic dendrites are pivotal in shaping the microstructure and performance of metallic alloys, especially in systems like Al-Cu alloys. These dendritic formations, which develop during solidification, play a substantial role in determining the mechanical, thermal, and corrosion-resistant properties of the material. In hypereutectic Al-Cu alloys, the presence of primary Al_2_Cu intermetallic dendrites contribute to the strength, hardness, and wear resistance of the alloy. However, oversized and coarse dendrites can have adverse effects, leading to diminished mechanical properties and reduced ductility. Wang et al. [91] utilized synchrotron X-ray high-speed radiography to explore the in situ breakup and refinement of Al_2_Cu intermetallic dendrites in a hypereutectic Al-35% Cu alloy during ultrasonic melt processing (UMP). The study demonstrated that acoustic cavitation and streaming flow induced by ultrasound are pivotal in breaking down the intermetallic dendrites. Notably, streaming flow serves two key functions: it directly acts on the intermetallic dendrites, and facilitates the transport of bubbles closer to the dendrites, as shown in Figure 8. These findings provide valuable insights into the fundamental mechanisms of dendrite refinement.

**Figure 9:**
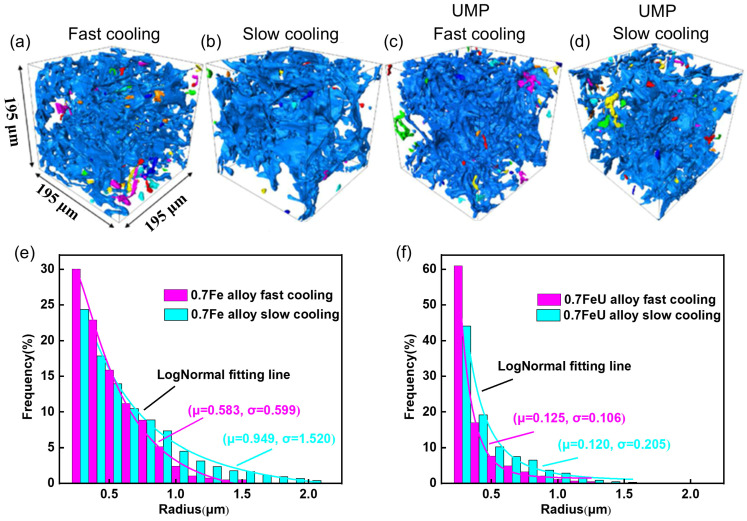
Three-dimensional morphology of the Fe-rich phase under different cooling rates: (**a**) 0.7 Fe with a fast cooling rate; (**b**) 0.7 Fe with a slow cooling rate; (**c**) 0.7 FeU with a fast cooling rate; (**d**) 0.7 FeU with a slow cooling rate, the different colors shown in (**a**–**e**) represent Fe-rich phases, but they correspond to distinct individuals. Panels (**e**,**f**) display the mean radius of the Fe-rich phase in 0.7 Fe and 0.7 FeU alloys [22].

Fe-rich phases are particularly important in aluminum alloys, such as Al-Fe alloys, as they significantly influence the material’s mechanical properties and performance. In these alloys, phases like AlFeSi and AlFe form during solidification, which in turn affects properties such as strength, ductility, and corrosion resistance. Furthermore, the cooling rate plays a crucial role in controlling the solidification structure, directly impacting these material characteristics. Zhao et al. [22] investigated the synergistic effects of ultrasound melt processing (UMP) and cooling rate on the microstructure of Al-Fe alloy systems. They found that increasing the cooling rate after UMP refines the α-Al grain structure and inhibits the growth of Fe-rich phases, Al_2_Cu, and pores. The 3D morphologies of Fe-rich phases and Al_2_Cu transition from coarse, irregular shapes to finer, more compact ones, with reductions in both local thickness and radius. Figure 9 shows the 3D morphology the Fe-rich phase under different cooling rates and mean radius of the Fe-rich phase. Faster cooling rates effectively suppressed pore growth, particularly in the 0.7 FeU alloy, where pores nearly disappeared. In the 1.2 Fe alloy, as the cooling rate increased from 0.1 °C/s to 1.5 °C/s, the number of Fe-rich phases increased while their size decreased, indicating that higher cooling rates promote nucleation and prevent excessive growth. This leads to a more uniform distribution of Fe-rich phases and enhanced mechanical properties. Additionally, these findings highlight the synergistic effects of UMP and cooling rate in refining microstructure and improving mechanical properties, suggesting that optimizing these parameters can significantly enhance material performance in industrial applications.

**Figure 10:**
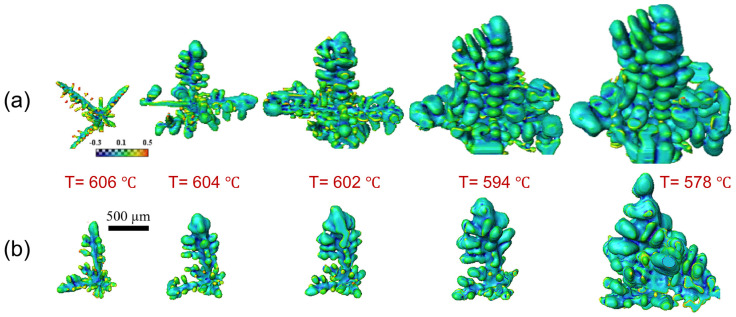
The growth and morphological evolution of individual Al dendrites under two conditions: without (**a**) and with (**b**) UMP [85].

Advancements in synchrotron X-ray technology have greatly enhanced data transmission speeds, facilitating real-time tomography with synchrotron X-ray sources [89,92,93]. Zhang et al. [85] conducted in situ tomography study to observe the phase fragmentation and growth dynamics of dendritic microstructures in an Al-15%Cu alloy during solidification, both with and without ultrasound treatment. As shown in Figure 10, the application of ultrasound for just a few seconds resulted in a dramatic increase in dendrite fragmentation, with a roughly 100% increase compared to that of untreated samples. This led to a significant reduction in the average grain size of the solidified samples, decreasing by 20–25%. As shown in Figure 10a, α-Al dendrites grew with four-fold crystallographic symmetry, with primary arms perpendicular to each other. Al dendrites, being face-centered cubic crystals, preferentially grow along low-index directions like <100>, where the solid–liquid interfacial energy is lowest. Secondary arms also predominantly grew along <100>, though slight deviations from symmetry occurred due to Cu addition, which disrupted the interfacial energy. Figure 10b illustrates that UMP did not change the crystal growth orientation, and Al dendrites retained their four-fold symmetry. The real-time, near-operando data collected provided invaluable 4D insights, which are crucial for validating numerical models and developing strategies to optimize UMP in industrial applications.

## 5. The Modeling and Simulation Methods for Complementing the Experimental Methods

Ultrasonic melt processing (UMP) is a technique that utilizes high-intensity ultrasound to induce complex, multiscale dynamics within liquid systems, occurring across both time and space. This process involves alternating acoustic pressures and bubble collapses that take place on extremely short timescales, ranging from microseconds to nanoseconds. Experimentally capturing these intricate dynamics is challenging due to their rapid and transient nature. Consequently, high-fidelity computational models are essential for gaining a deeper understanding of the physical mechanisms driving UMP. These simulations offer three-dimensional visualizations that highlight the interactions between sound waves, bubbles, and the solid phase—interactions that are difficult to observe directly in experiments. Computational modeling for UMP is divided into four categories: analytical, numerical, molecular dynamics, and interaction models. Each provides a unique perspective on the governing equations, with specific strengths and limitations, collectively enhancing our understanding of the process.

### 5.1. Analytical Model

**Figure 11:**
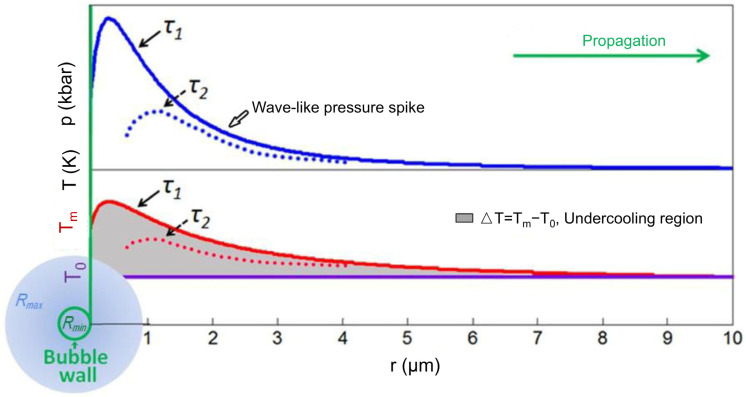
A schematic illustrating the temperature (red curves) and pressure (blue curves) profiles near an imploding bubble is presented. The bubble is characterized by two distinct radii: Rmax, the maximum radius just before implosion, and Rmin, the minimum radius immediately after implosion. The pressure-induced undercooling (*Tm* − *T*_0_) is represented by the gray shaded region beneath the temperature profile [86].

Analytical models rely on mathematical formulations to represent physical processes, offering quick results without the need for extensive numerical computations. This makes them particularly suited for real-time decision-making and rapid assessments. For instance, Huang et al. [86] developed an analytical model to estimate cavitation and the distribution of grain sizes in an Al-Cu alloy system. The nucleation number at a single implosion event is determined by the following:

(1) $$N_{i}=\int_{\tau }^{.}\int_{V}^{.}I_{nuc}\left(r,\tau \right)dVd\tau$$

where $N_{i}$ represents the nucleation count, with the subscript i denoting the bubble size; $I_{nuc}$ is the nucleation rate within the undercooled volume V at time τ. The nucleation rate $I_{nuc}$ is given by the following [94,95]:

(2) $$I_{nuc}=I_{0}exp\left(-\frac{{∆G}^{*}\left(T\right)\cdot f\left(\theta \right)}{kT}\right)\cdot exp\left(-\frac{Q}{kT}\right)$$

where k is the Boltzmann constant; $I_{0}=nkT/h$; n is the atom number per unit volume; and h is the Planck constant. Additionally, ${∆G}^{*}\left(T\right)$ represents the energy barrier for forming a critical nucleus, calculated as follows:

(3) $${∆G}^{*}\left(T\right)=\frac{16\pi }{3}\frac{\sigma_{sl}^{3}T_{m}^{2}\cdot V_{mol}^{2}}{{∆H}^{2}\left(T_{m}-T\right)}$$

where $T_{m}$ is the melting temperature; ∆H is the phase change enthalpy; $V_{mol}$ is the molar volume of the solid phase; $\sigma_{sl}$ is the solid–liquid interfacial energy. While these parameters are typically constant under atmospheric pressure, they are treated as pressure dependent. The pressure dependence $T_{m}$ is approximated for pure aluminum using the best Simon fit: [96],

(4) $$T_{m}=T_{m0}{\left(\frac{p}{60.49}+1\right)}^{0.531}$$

where $T_{m0}$ is the reference temperature. The undercooling at a specific location is written as follows, more details can be found in Figure 11:

(5) $$∆T=T_{m}\left(p\right)-T_{0}$$

The local melt temperature is denoted as $f\left(\theta \right)$. The phase change enthalpy ∆H in Equation (3) can be derived from the Clausius–Clapeyron equation, which is written as follows:

(6) $$\frac{dlnT_{m}}{dp}=\frac{∆V}{∆H}$$

By rewriting this, we obtain the following:

(7) $$∆H=∆V\cdot \frac{dp}{dlnT_{m}}=∆V\cdot T_{m}\frac{dp}{dT_{m}}$$

Here, $∆V=V_{s}-V_{l}$ is the molar volume difference in the solid ($V_{s}$) and liquid ($V_{l}$), which is treated as a constant. The term $\frac{dp}{dT_{m}}$ is calculated by differentiating the melting point expression. The interfacial energy $\sigma_{sl}$ in Equation (3) is expressed as a function of the phase change enthalpy:

(8) $$\frac{\sigma_{sl}}{\rho_{s}^{2/3}}=Cm^{1/3}∆H$$

where m is the atomic mass and C is a constant. The interface energy is determined by comparing it to a reference value:

(9) $$\frac{\sigma_{sl}}{\sigma_{sl0}}={\left(\frac{\rho_{s}}{\rho_{s0}}\right)}^{\frac{2}{3}}\cdot \frac{∆H}{{∆H}_{0}}$$

where $\rho_{s}$ is the solid density; $\sigma_{sl0}$, $\rho_{s0}$, and ${∆H}_{0}$ are the reference solid–liquid interface energy, the solid density, and the phase change enthalpy, respectively.

The activation energy Q in Equation (2) represents the energy required for an atom to diffuse across the liquid–solid interface, which is written as

(10) $$Q=kTln\left(\frac{\eta V}{hN}\right)$$

where N is the Avogadro constant; V is the molar volume of the liquid; η is the absolute viscosity, which follows the Arrhenius equation [97]:

(11) $$\eta =\eta_{0}exp\left(\frac{Q_{a}+P∆V_{a}}{RT}\right)$$

where $Q_{a}$ and $∆V_{a}$ are the activation energy and the activation volume, respectively, and $\eta_{0}$ is a pre-potential viscosity factor. However, since data for these parameters specific to the Al-18 wt.% Cu melt have not been reported, the fitted data from pure aluminum melt in reference [98] were used for approximation in Equation (11). The explicit expression for η is

(12) $$\eta =0.29\cdot exp\left(\frac{12000+1\times 10^{-6}\cdot p}{8.31447\cdot T}\right)$$

In Equation (2), $f\left(\theta \right)$ represents the geometric factor, generally defined as follows [35]:

(13) $$f\left(\theta \right)=\frac{{\left(1-\cos\left(\theta \right)\right)}^{2}\cdot \left(\cos\left(\theta \right)+2\right)}{4}$$

where θ is the wetting angle. The wetting angle *θ* is influenced by various factors, including substrate type, properties, and solid–liquid interfacial energy. Typically, the value of θ is treated as a constant, independent of solute content, as carried out in other studies [95,99,100]. Here, the wetting angle is considered as angle between the alumina and the aluminum melt. For polycrystalline aluminum with a surface roughness of 720 Å, the wetting angle is approximately constant at 86° across different temperatures [101].

From Equations (1)–(12), $I_{nuc}$ is a function of both the melt pressure p and the melt temperature T, and the nucleation number $N_{i}$ in Equation (1) is computed by determining p, which represents the pressure wave profile in the melt surrounding the imploding bubble.

To estimate the shock pressure, the characteristic method [102] based on the Kirkwood–Bethe hypothesis was applied. This method assumes that an invariant quantity $Y=r\left(h+u^{2}/2\right)$ propagates along outward “characteristic” curves, with the characteristic velocity given by c+u, where r is the radial distance to the bubble center, and h, c, and u represent the local enthalpy, velocity, and speed of sound in the liquid, respectively. The characteristics of the curves, along with the velocity u and the pressure $p_{c}$ along these curves, are governed by the following equations [103]:

(14) $$\frac{du}{dt}=\frac{1}{r\left(c-u\right)}\left[\left(c+u\right)\frac{Y}{r}\right]-2c^{2}u$$

(15) $$\frac{{dp}_{c}}{dt}=\frac{n\left(p_{c}+B\right)}{rc\left(c-u\right)}\left[2cu^{2}-\frac{c+u}{Y}\right]$$

(16) $$\frac{dr}{dt}=c+u$$

These equations are solved by integrating the velocity and pressure derivatives along the characteristic curves, starting from the bubble wall, as derived from the Gilmore equation [103]:

(17) $$R\frac{dU}{dt}\left(1-\frac{U}{C}\right)+\frac{3}{2}U^{2}\left(1-\frac{U}{3C}\right)=H\left(1+\frac{U}{C}\right)+\frac{RU}{C}\frac{dH}{dR}\left(1-\frac{U}{C}\right)$$

In these equations, B and n are empirical constants which depend on the type of the liquid; σ, μ and γ represent the surface tension, viscosity, and the gas polytropic exponent of the liquid, respectively. R and U refer to the radius and velocity of the bubble, respectively. The maximum bubble diameter (the largest size of the oscillating bubble) was measured from synchrotron X-ray images and used as a boundary condition to regress the Gilmore Equation (17). This regression allowed for the determination of the velocity and pressure derivatives needed for Equations (14)–(16).

The analytical model described above is highly effective for predicting the behavior of a single bubble. However, it requires further refinement to address multi-bubble systems. This is because the model attributes the implosion of a single bubble primarily to acoustic pressure, whereas in a multi-bubble system, implosions are often triggered by a chain reaction caused by the shock wave released after the initial bubble implodes. The numerical simulation method discussed below provides a more comprehensive approach to addressing this phenomenon.

### 5.2. Numerical Modeling

#### Acoustic Pressure

The acoustic pressure distribution in space and time through a liquid medium can be obtained by solving the wave equation [104]. If linear wave propagation is assumed, the shear stress is neglected and the fluid is incompressible, which is corrected for liquids, the wave equation has the following form:

(18) $$\nabla \left(\frac{1}{\rho }\nabla p\right)-\frac{1}{\rho c^{2}}\frac{\partial^{2}p}{\partial t^{2}}=0$$

where ρ is the fluid density, C is the speed of sound in the liquid medium, p is the acoustic pressure. With the assumptions of linearity of the sound wave, neglecting the shear stresses and incompressible liquid medium, t is time harmonic, i.e.,

(19) $$p\left(r,t\right)=p\left(r\right)e^{i\omega t}$$

The space-dependent part of the pressure $p\left(r\right)$ is the solution of the Helmholtz equation,

(20) $$\nabla \left(\frac{1}{\rho }\nabla p\right)-\frac{\omega^{2}}{\rho c^{2}}p=0$$

where r is the distance to the probe and *ω* is the angular frequency of the ultrasound.

As the distance from the probe increases, the intensity decreases according to an area into which it is spread in a conical shape.

**Figure 12:**
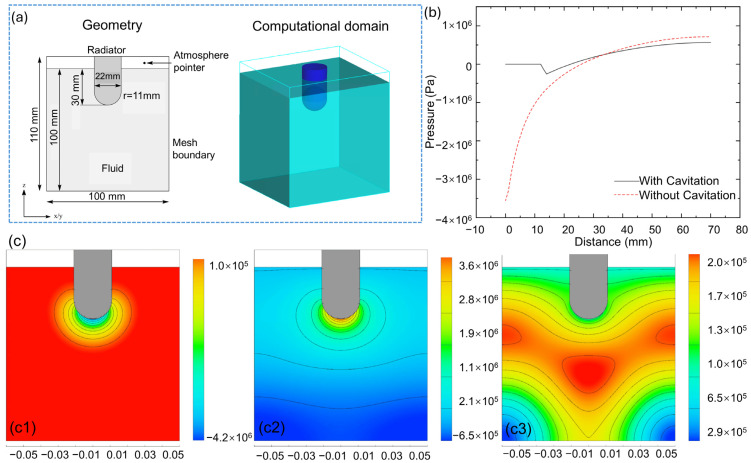
(**a**) Geometry and computational domain; (**b**) pressure profiles along the Z axis; (**c**) development and propagation of sound waves in aluminum A356 at different stages: (**c1**) 3 μs, (**c2**) 25.8 μs, and (**c3**) 62.5 μs [76].

Riedel et al. [76] conducted a series of simulations in pure aluminum. The computational domain, model, and pressure profiles are shown in Figure 12a,b, while the pressure initiation and distribution through aluminum arere shown in Figure 12c. At t = 3 μs, negative pressure increased by several MPa below the radiator face and propagated in the –z direction, followed by the development of positive pressure in the same direction. The spherical geometry of the radiator also facilitated propagation in the xy direction. The peak pressures, ranging from 4.2 MPa to 4.5 MPa, were observed at the center of the radiator faces, with pressure intensity decreasing exponentially as the distance increased. Furthermore, reflected waves from the sidewalls caused a slower pressure decrease in these regions due to interference, which was influenced by the simulation boundary conditions.

Rashwan et al. [104] used the computational acoustics module within COMSOL Multiphysics 2022 r1 to explore the influence of acoustic and geometric parameters, such as ultrasonic frequency, acoustic intensity, and reactor scaling, on reactor performance. The findings indicate that increasing the ultrasound frequency alters the periodic time and carrier signal wavelength, affecting the phase of reflected waves from hard wall boundaries. The study highlights the highest cavitation energy within the 60 to 80 kHz frequency range, corresponding to the maximum magnitude of negative pressure. Cavitation energy analyses were conducted at a 20 kHz frequency and 36 W input power. Additionally, altering the transducer probe depth had a minimal impact on the acoustic pressure field. Consequently, they proposed increasing ultrasound input power for frequencies below 60 kHz.

### 5.3. Ultrasonic Bubble Dynamics

Understanding bubble dynamics is crucial for optimizing UMP. Bubbles formed during agitation methods like sonication aid in material fragmentation or exfoliation by providing mechanical shearing and dispersion. Additionally, fluid flow patterns induced by bubble formation and collapse enhance mixing and dispersion, while cavitation effects from bubble collapse aid in material breakdown. Interactions between bubbles and the substrate surface influence material adhesion and bonding. Thus, studying bubble dynamics informs the optimization of process parameters for efficient UMP techniques.

Moving onto modeling approaches, the Navier–Stokes equations provide the foundation for fluid mechanics at the macroscopic scale, enabling the tracking of bubble interfaces through techniques such as the Volume of Fluid (VOF) and Level Set methods. At the microscopic level, molecular dynamics simulations allow for the study of bubble formation and implosion, which are critical for shock wave generation. Finally, the lattice Boltzmann method (LBM) serves as a bridge between the macroscopic and microscopic scales, offering an efficient way to simulate bubble dynamics and complex interfaces [105]. With its mesoscopic resolution, LBM captures both macroscopic flow behaviors and microscale phenomena, providing high computational efficiency.

#### 5.3.1. Modeling Based on Navier–Stokes

The Navier–Stokes (NS) equations govern the motion of fluid particles and the distribution of velocity, pressure, and density in a continuous medium. These equations are derived from the fundamental principles of conservation of mass, momentum, and energy, making them the cornerstone of computational fluid dynamics (CFD) at the macroscopic scale [106].

The mass conservation (continuity) equation is given by the following:

(21) $$\frac{\partial \rho }{\partial t}+\nabla \cdot \left(\rho u\right)=0$$

where *ρ* is the density; *t* is the time; and *u* denotes the velocity vector of the flow field.

The momentum balance equation including the acoustic radiation force term can be written as

(22) $$\frac{\partial \rho u}{\partial t}+\rho u\cdot \nabla u=-\nabla p+\rho g+\nabla \cdot \left[\eta \left(\nabla u+\nabla^{T}u\right)-\left(\frac{2}{3}\eta -\eta_{b}\right)\nabla \cdot uI\right]+F$$

where *p* is the pressure; ***g*** is the gravitational acceleration; *ρ* is the density; *η* is the viscosity of the fluid with the non-acoustic situation; $\eta_{b}$ is the dilatational viscosity; I is the identity tensor; F is the external force, and it can stand for different forces in different models.

The energy conservation equation in terms of the total energy is

(23) $$\frac{\partial \rho E}{\partial t}+\nabla \cdot \left(\rho uE\right)+\nabla \cdot \left(up\right)=-\nabla \cdot q+\nabla \cdot \left(\tau \cdot u\right)+\rho r+(\rho g+F)\cdot u$$

where $E=e+\frac{u^{2}}{2}$ is the specific total energy (total energy per unit mass), and e is the specific internal energy, and $\frac{u^{2}}{2}$ stands for the specific kinematic energy, q is the specific heat source, τ is the viscous stress tensor.

The Navier–Stokes equations provide the governing equations for fluid motion, while interface tracking techniques such as the Volume of Fluid (VOF) and Level Set techniques complement these equations by allowing for the accurate representation of interfaces and boundaries within the fluid domain. Together, they form a comprehensive framework for modeling and simulating fluid flow phenomena, including those involving bubble dynamics in applications like UMP.

In the VOF method, the phase interface (air–water interface) is characterized by the fraction of liquid volume in each cell [107]. The movement of this interface is governed by solving the advection equation for the liquid volume fraction (*ψ*) [108]:

(24) $$\frac{\partial \psi }{\partial t}+u\cdot \nabla \psi =0$$

Here, *ψ* = 1 represents the fluid phase, *φ* = 0 denotes gas, and *ψ* ranges between 0 and 1 for the mixture phase.

The density and viscosity of the mixture phase are interpolated as follows:

(25) $$\rho =\psi \rho_{l}+(1-\psi )\rho_{g},\;\eta =\psi \eta_{l}+(1-\psi )\eta_{g}$$

where subscripts l and g denote the liquid phase and the gas phase, respectively.

The external force in the momentum and energy equations includes the surface tension force, $F_{\sigma }$, which can be obtained by the continuum surface force model $F_{\sigma }=\sigma \kappa \nabla \psi$, where κ is the surface curvature, which can be calculated from the liquid volume fraction through

(26) $$\kappa =-\nabla \cdot \left(\frac{\nabla \psi \;}{\left|\nabla \psi \right|}\right).$$

**Figure 13:**
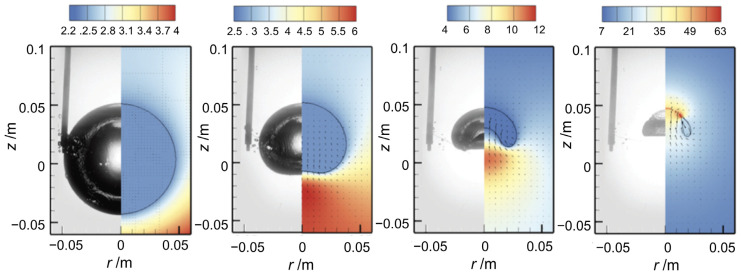
Comparison of the bubble collapse results from the experiment (**left**) and the numerical simulation (**right**) at $t^{*}$ = 1.41, 2.52, 2.84, 3.00. The color contour represents the pressure field (in kPa) [109].

Figure 13 illustrates the bubble collapse process, comparing experimental and numerical results by Liu et al. [109] employing the Volume of Fluid (VOF) method for bubble shape tracking. The simulation captures the collapse initiation at $t^{*}$ = 1.41, marked by the bubble’s maximum volume before collapse begins. Gravity induces a downward hydrostatic pressure gradient around the bubble, causing faster collapse of its lower part due to higher pressure. A high-pressure zone forms beneath the bubble, accelerating the lower surface collapse and generating a rapid upward jet (nondimensional speed ~1.8) penetrating the bubble by $t^{*}$ = 3.00. Overall, the simulation closely aligns with experimental observations, except for a slightly higher jet head position at $t^{*}$ = 2.84.

**Figure 14:**
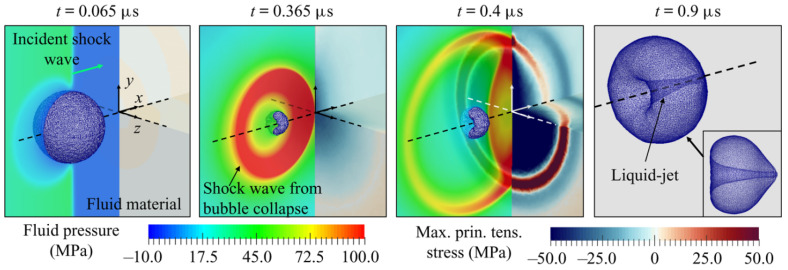
The dynamics of bubble deformation, transient fluid pressure, and the maximum stress both within the material and on its surface during shock-induced bubble collapse near BegoStone, (a type of strengthened gypsum) [110].

Figure 14 presents four snapshots of solution states depicting shock-induced bubble collapse near BegoStone, which is a kind of strengthened gypsum, as studied by Cao et al. [110]. The Level Set method is used to track the gas–bubble interface. The impact of the incident shock wave on the proximal (left) side initiates the bubble’s collapse. As the bubble reaches its minimum volume, a shock wave is generated. During the non-spherical collapse, a liquid jet forms and penetrates the bubble. Simultaneously, the reflected wave impacts the bubble from the distal (right) side, further accelerating its collapse. When the emitted shock wave reaches the material surface, part of its energy is reflected. This reflected wave, which is compressive and of comparable amplitude to the incident shock, enhances the bubble’s collapse.

#### 5.3.2. Modeling Based on Rayleigh–Plesset Equation

**Figure 15:**
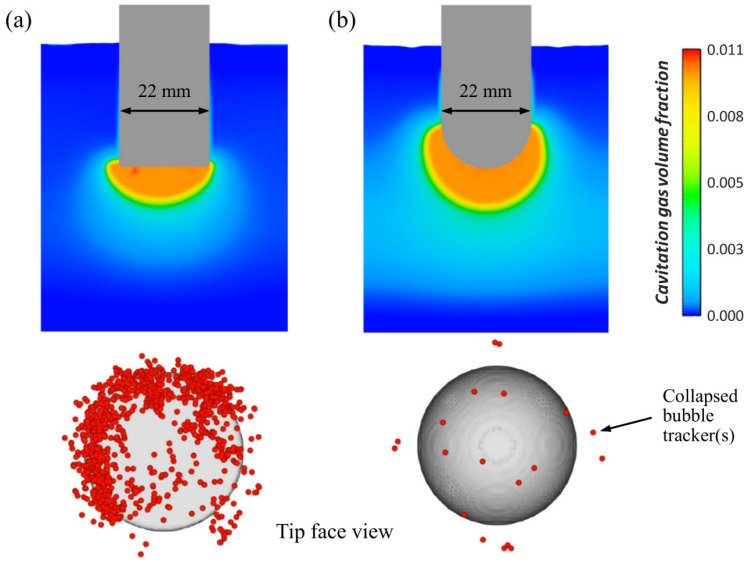
Cavitation zone development and the corresponding bubble collapse activity after 0.01 s of UMP in A356: (**a**) flat radiator tip and (**b**) spherical radiator tip [69].

To accurately capture cavitation zones, several commercial flow dynamics software packages, such as ANSYS Fluent 2024 r2 and FLOW-3D 2024 r1, have developed specialized macroscopic cavitation models, primarily based on solving the Rayleigh–Plesset equation. Riedel et al. [76] presented cavitation development results for both spherical and flat radiator tips, as illustrated in Figure 15. They also analyzed the collapse activity associated with different radiator shapes, using FLOW-3D to track collapsed bubbles via void particles. Cavitation primarily occurs in the region directly beneath the radiator, where the highest-pressure peaks are located, creating optimal conditions for cavitation. Interestingly, while the spherical radiator produces a larger volume of cavitation gas, the flat radiator, though having a smaller cavitation zone, exhibits significantly higher collapse activity compared to the spherical radiator.

### 5.4. Molecular Dynamics Modeling

**Figure 16:**
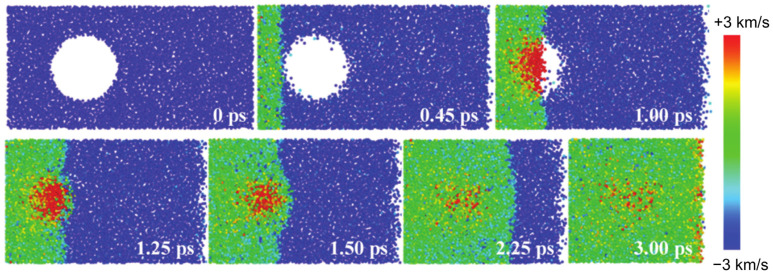
A sequence of snapshots illustrating the propagation of a shock wave through water for a bubble diameter of D = 5.0 nm at an impact velocity of up = 3.0 km/s. These snapshots capture the dynamics of the shock wave as it travels through the water, highlighting key stages of the interaction and bubble behavior [114].

The molecular dynamics (MD) model is a computational technique used to simulate the behavior of atoms and molecules in a system over time. In the context of bubble dynamics, MD simulations are employed to study the formation, evolution, and collapse of bubbles in a liquid medium at the molecular level:

In molecular dynamics (MD) simulations of bubble dynamics, interactions between atoms and molecules are governed by interatomic potential energy functions, such as Lennard–Jones or Morse potentials, which determine the forces between particles [111,112,113]. Zhan et al. [114] used MD simulations to examine the implosion of microscopic bubbles under compression by varying impact velocities of water molecules in Figure 16. Key parameters, such as shock wave velocity, bubble implosion time, and shock pressure induced by bubble implosion, were quantified. The results revealed that, with increasing impact velocities, the bubble collapse times shortened and micro-jet velocities increased. Additionally, following bubble implosion, secondary water hammer shocks and elevated shock pressures were simulated. At an impact velocity of 1.0 km/s, a phase transition to the ice-VII structure in the water molecules was detected. However, these simulations come with significant limitations. One major constraint is the high computational cost, which makes it challenging to model multiple bubbles simultaneously. Additionally, simulating large micron-scale areas is not feasible due to the intensive computational requirements. These limitations restrict the scalability of molecular dynamics simulations, making it difficult to study larger systems or longer timeframes.

### 5.5. Interaction Modeling

An interaction model provides valuable insights into the interplay between bubbles and solidifying metals, uncovering their effects on crystal formation, solute distribution, and defect generation. By analyzing bubble-induced convection, the model helps reduce segregation and improve structural uniformity. It also simulates bubble collapse near interfaces, addressing localized remelting and defect prevention. Incorporating surface tension dynamics, multiphysics interactions, and process parameters such as ultrasonic treatment, the model enhances process design and material quality, advancing the understanding and control of metallic solidification processes.

**Figure 17:**
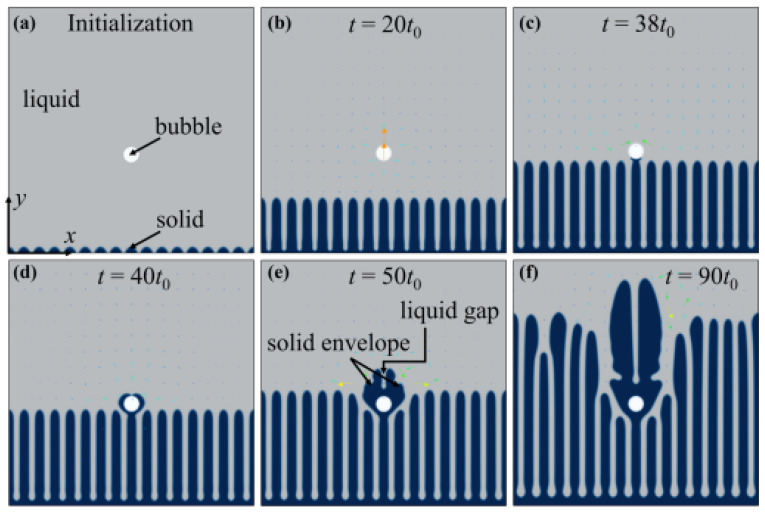
Typical simulation results obtained using PFLB, illustrating the interaction between an existing bubble and a cellular interface over time: (**a**) *t*_0_, (**b**) 20 *t*_0_, (**c**) *38 t*_0_, (**d**) 40 *t*_0_, (**e**) 50 *t*_0_ and (**f**) 90 *t*_0_ [115].

Zhang et al. [115] addressed the complexities of simulating solid–liquid–gas interactions during solidification, with a particular focus on intricate phase boundaries, bubble dynamics, and the significant density differences between the liquid and gas phases. A hybrid phase-field lattice Boltzmann (PFLB) method, coupled with a parallel adaptive mesh refinement (Para-AMR) algorithm, was introduced to model the interactions between gas bubbles and the advancing solidification front. The phase-field technique was applied to describe solid growth and bubble dynamics, while the lattice Boltzmann model captured melt flow and bubble motion. Experimental comparisons validated the model for various solid/liquid interface morphologies, including planar, cellular, and dendritic structures. The findings indicate that bubble impacts on the solidification process depend on interface shape, bubble size, and spatial relationships between bubbles and dendritic tips. Two classic interaction modes, namely engulfment and entrapment, were analyzed, with solute redistribution identified as the key differentiating factor. Additionally, the behavior of multiple rising deformed bubbles interacting with dendritic arrays was investigated, as shown in Figure 17. Such a PFLB method combines the strengths of phase-field modeling and the lattice Boltzmann approach. It naturally captures interface evolution, including topology changes like merging and splitting, without explicitly tracking interfaces. PFLB is well suited for simulating multiphase flows and complex geometries, offering efficient coupling of hydrodynamics and interface dynamics. However, its limitations include high computational costs due to its fine grid resolution requirements and challenges in parameter tuning for specific materials. Additionally, it may struggle with extreme density or viscosity contrasts, requiring careful numerical treatment to maintain stability and accuracy.

## 6. Summary and Outlook

Over the past five decades, significant progress has been made in the field of ultrasonic melt processing, particularly in understanding the effects of ultrasound on liquid metal structures and solidification dynamics. Despite these advancements, the industrial adoption of ultrasonic melt processing remains limited. To address this gap, foundational research must tackle several key practical questions: What is the optimal melt flow rate to maximize treatment efficiency while minimizing energy consumption, costs, and system complexity? What are the ideal frequency and acoustic power settings to improve treatment outcomes? Where should the ultrasonic power source be located within the melt transfer system to optimize its interaction with the melt pool configuration? The introduction of powerful in situ characterization tools, such as high-speed imaging and synchrotron X-rays, has accelerated research, allowing detailed exploration of phenomena such as cavitation, acoustic streaming, and the refinement of microstructures in various metal alloys systems. Combining advanced experimental techniques with robust numerical simulations, researchers have achieved a more accurate depiction of the interactions between ultrasonic waves and liquid metals, shedding light on critical mechanisms such as grain refinement and porosity reduction. However, challenges remain, particularly in capturing real-time dynamics at the microscopic scale during rapid solidification and under extreme conditions. Further integration of cutting-edge methods like machine learning and multi-physics simulations is necessary to simulate and predict complex behaviors with higher fidelity. As computational capabilities continue to advance, these approaches are expected to resolve long-standing scientific and technological issues in ultrasonic melt processing, paving the way for more efficient and precise control in industrial applications.
